# Optimal exercise dose and type for improving sleep quality: a systematic review and network meta-analysis of RCTs

**DOI:** 10.3389/fpsyg.2024.1466277

**Published:** 2024-10-03

**Authors:** Li Li, Chunxiao Wang, Dandan Wang, Hua Li, Shuai Zhang, Yuanchun He, Ping Wang

**Affiliations:** ^1^Postgraduate School, University of Harbin Sport, Harbin, China; ^2^School of Sport Science and Health, University of Harbin Sport, Harbin, China; ^3^Physical Education Department of Xiamen University, Xiamen, China

**Keywords:** exercise, sleep, sleep quality, randomized controlled trial, network meta-analysis

## Abstract

**Background:**

A substantial amount of research has explored the intricate relationship between exercise and sleep quality, consistently confirming that exercise can effectively enhance sleep quality. Nevertheless, previous studies have yet to conclusively determine which specific exercise program is most efficacious in improving sleep quality. To address this gap, the present study systematically evaluated the differential effects of various types of exercise, as well as exercise dosages (including duration, intervention period, frequency, and intensity), on sleep outcomes using a network meta-analysis approach. This endeavor aims to provide evidence-based support for the development of scientifically effective exercise programs tailored to improve sleep quality.

**Methods:**

Through the Web of Science, PubMed, Cochrane Library, Embase, and Scopus databases, we conducted a search for randomized controlled trials investigating the effects of exercise interventions on sleep, with a search cutoff date of April 30, 2024. We rigorously selected the literature according to the PICOS principle, and two independent researchers extracted the data. We would like to change this passage to: Bias risk assessment was conducted using the RevMan 5.4 software, and traditional meta-analysis and network meta-analysis were performed using Stata 17.0 software to generate forest plots, network evidence plots, and funnel plots. Furthermore, we adopted the surface under the cumulative ranking curve (SUCRA) to evaluate and rank the intervention effects of different exercise types and dosages on sleep quality. To verify the robustness of our study results, we performed a sensitivity analysis using the leave-one-out method.

**Results:**

The study strictly adhered to the PRISMA guidelines and included 58 RCT papers with a total of 5,008 participants. The network meta-analysis revealed significant variations in the impact of exercise frequency on sleep outcomes when compared to the control group. Interventions of 1–2 times per week [SMD = −0.85, 95% CI (−1.43, −0.26)], 3 times per week [SMD = −0.45, 95% CI (−0.80, −0.11)], and 4 times per week [SMD = −1.09, 95% CI (−1.92, −0.26)] demonstrated the most notable effects. Interventions lasting ≤30 min and 60–65 min were significantly more effective than the control group, with ≤30 min proving significantly more effective than 40–55 min [SMD = 0.75, 95% CI (0.01, 1.49)]. Interventions lasting 9–10 weeks [SMD = −1.40, 95% CI (−2.37, −0.44)], 12–16 weeks [SMD = −0.55, 95% CI (−0.90, −0.20)], and ≥ 24 weeks [SMD = −0.71, 95% CI (−1.31, −0.10)] were all significantly more effective than the control group. Additionally, the 9–10 weeks intervention period was found to be significantly more effective than the 6–8 weeks period [SMD = −1.21, 95% CI (−2.37, −0.04)]. Furthermore, interventions of moderate intensity [SMD = −1.06, 95% CI (−1.52, −0.61)] and high intensity [SMD = −1.48, 95% CI (−2.55, −0.40)] exercise interventions yielded significantly greater benefits compared to the control group. Specifically, high intensity interventions [SMD = −1.97, 95% CI (−3.37, −0.56)] and moderate intensity [SMD = −1.55, 95% CI (−2.57, −0.54)] exercise interventions were found to be significantly more effective than moderate-high intensity interventions. In terms of exercise types, aerobic exercise [SMD = −0.56, 95% CI (−0.86, −0.27)], traditional Chinese exercises [SMD = −0.57, 95% CI (−0.97, −0.18)], and combined exercise [SMD = −0.99, 95% CI (−1.66, −0.32)] interventions all produced significant improvements compared to the control group. The study determined that the most effective combination of exercise prescription elements for enhancing sleep quality includes a frequency of 4 times per week (SUCRA = 84.7), a duration of ≤30 min (SUCRA = 92.2), a period of 9–10 weeks (SUCRA = 89.9), and high-intensity (SUCRA = 92.9) combined exercise (SUCRA = 82.7).

**Conclusion:**

The current evidence indicates that combined exercise with a frequency of 4 times per week, a duration of ≤30 min, a period of 9–10 weeks, and high intensity is most effective for improving sleep quality. Nevertheless, due to the limited number of studies included, further research is needed to enhance the reliability of the findings.

**Systematic review registration:**

https://www.crd.york.ac.uk/prospero/, identifier: CRD42024555428.

## 1 Introduction

The quality of sleep serves as a crucial physiological metric that evaluates an individual’s sleep status, incorporating subjective perception and objective sleep parameters (Ohayon et al., 2017). Adequate sleep is a fundamental component of a healthy lifestyle, as it can bolster memory consolidation, enhance attention and immune function (Moosavi-Movahedi et al., 2021), efficiently replenish expended energy from daily activities (Chandrasekaran, 2020), and promote overall well-being (Vandekerckhove and Wang, 2018). Furthermore, it plays a critical role in safeguarding cardiovascular health (Plante, 2006; Holfinger et al., 2021); regulating bodily processes, and sustaining optimal brain function (Plante, 2006; Pickersgill et al., 2022). Due to the crucial role of sleep in regulating and preserving human health, the quality of sleep has emerged as a key indicator for evaluating health status and overall quality of life (Dzierzewski et al., 2021). Presently, concerns regarding sleep quality have garnered widespread attention in society.

Sleep issues are present across all age groups, from adolescents to middle-aged and older adults. Relevant survey data show that among adolescents, about two-thirds are observed to have less than 6 h of sleep (Ohida et al., 2004; Paruthi et al., 2016). Furthermore, globally, over one-third of adults face varying degrees of sleep disturbances (Panel et al., 2015; Liu, 2016; Galinsky et al., 2018). Among the elderly population, the problem is even more pronounced, with up to 50% of older adults experiencing difficulty falling asleep or maintaining sleep (Paruthi et al., 2016; Schroeck et al., 2016). Epidemiological studies consistently indicate that sleep problems tend to intensify with increasing age (Almondes et al., 2021), a decline in sleep quality not only hampers individual learning capabilities but also compromises immune function (Dzierzewski et al., 2021). In addition, poor sleep quality is not only associated with a heightened susceptibility to chronic illnesses and an elevated likelihood of suicidal behaviors (Mikula et al., 2020; Bjorvatn et al., 2021), but also places a substantial financial strain on both families and society. For instance, in Canada, sleep-related issues lead to an estimated loss of $484 million (Streatfeild et al., 2021), while in Australia, the economic impact of sleep disorders is calculated at $35.4 billion (Chaput et al., 2022). This underscores the importance of addressing sleep quality as a critical public health concern that requires collective action on a global scale.

Pharmacological treatment, as a conventional means of improving sleep, although effective, is associated with potential dependency, a higher risk of drug-related mortality (especially in cases of overdose) (Bushnell et al., 2022), and uncertainty in therapeutic outcomes (Sys et al., 2020). These factors have driven the search for safer and more efficient non-pharmacological alternatives (Morrison et al., 2022). Non-pharmacological interventions, particularly exercise interventions, are gradually becoming the preferred choice for improving sleep quality due to their low risk and significant effects (Rios et al., 2019). Physical activity, sport, and exercise are terms often used interchangeably in research (Chennaoui et al., 2015). Physical activity refers to any form of body movement that results in energy expenditure, encompassing all activities of daily life, whether professional, household, or recreational (Bull et al., 2020). Sport typically refers to competitive physical activities. Exercise is a component of physical activity; it is planned, structured, and characterized by its frequency, intensity, and duration (Chennaoui et al., 2015). It includes aerobic exercises, resistance training, jogging, dancing, Tai Chi, Baduanjin, etc., with specific types of exercise depending on an individual’s physical capacity (Hasan et al., 2022). Research has shown that sustained engagement in exercise has a notable positive impact on sleep quality. For instance, a 12-week moderate-intensity exercise program implemented by Tseng et al. demonstrated significant improvements in sleep quality. Furthermore, the study demonstrated a notable impact on enhancing sleep quality among elderly individuals (Tseng et al., 2020). Additionally, Siu’s investigation revealed that both Tai Chi and traditional sports activities were effective in improving sleep quality, with lasting effects for up to 24 months (Siu et al., 2021). Although meta-analyses have assessed the therapeutic effects of exercise on sleep quality and generally recognized the positive impact of exercise in improving sleep quality (Banno et al., 2018), there are still certain limitations in existing studies. Firstly, previous research has focused on the immediate effects of exercise on short-term sleep (such as the following day), and there is a lack of in-depth research on the impact of long-term exercise programs on sleep quality (Stutz et al., 2019). Secondly, studies have mostly concentrated on the assessment of a single or a few exercise doses (such as exercise type, duration of intervention), lacking a comprehensive evaluation of the integrated effects of exercise types and dosages. For example, Xie et al. only analyzed the impacts of exercise type and intervention duration on sleep quality (Xie et al., 2021), which limits our comprehensive understanding of the effects of exercise types and dosages. Furthermore, due to the methodological limitations of traditional meta-analyses, while they can confirm the positive impact of exercise on sleep quality (Banno et al., 2018), there is still a lack of evidence to prove which types of exercise and dosages are most effective in enhancing sleep quality. There is a need for systematic reviews and meta-analyses of randomized controlled trials (RCTs) in this field (Kreutz et al., 2019).

Network meta-analysis, implemented within the Bayesian framework with the aid of Markov Chain Monte Carlo simulation technology (Moher et al., 2015; Vats et al., 2019), is an advanced statistical method that can assess and compare the efficacy differences among multiple interventions (Lei et al., 2022). One of the advantages of network meta-analysis is its ability to integrate direct and indirect comparative evidence from randomized controlled trials into a network evidence map, allowing not only direct but also indirect evaluations of intervention effects (Tonin et al., 2017). Furthermore, it can compare and rank the effects of different interventions to identify the optimal intervention (Kodama et al., 2021). Compared to traditional meta-analysis, network meta-analysis demonstrates significant advantages in assessing precision (Cipriani et al., 2013). To our knowledge, no study has yet explicitly identified which combination of exercise type and dosage can most effectively improve sleep quality. Therefore, this study utilizes the network meta-analysis method, combined with data from randomized controlled trials, to analyze the differences in the effects of exercise on improving sleep quality from the perspective of exercise type and dosage, thus providing evidence-based support for the development of scientific and effective exercise programs.

## 2 Materials and methods

This study, adhering to the PRISMA guidelines for reporting systematic reviews and meta-analyses (Hutton et al., 2015), has been registered on the international platform PROSPERO with the registration number CRD42024555428.

### 2.1 Literature search

A thorough review of the literature was carried out by searching five databases, including Web of Science, PubMed, Embase, Cochrane Library, and Scopus, with a search timeframe up to April 30, 2024. Three sets of keywords were utilized to enhance the comprehensiveness of the search: ①exercise, physical activity, sport, tai chi, aerobic exercise, walking, pilates, yoga, qi gong, resistance exercise, combined exercise; ② insomnia, sleep disorder, sleep complaint, sleep disturbance, sleep quality, sleep problem; ③ intervention, Randomized Controlled Trial (RCT), experiment, trial. Boolean logical operators were used to connect the keywords. In addition to this, the reference lists of the retrieved articles were reviewed to identify potentially relevant studies. Additionally, a literature trace was performed by examining the included studies and their reference lists from published systematic reviews and meta-analyses to ensure the thoroughness of the search.

Taking the Web of Science as an example, the search strategy is as shown in the Table 1.

**Table 1:** Research retrieval methods based on Web of Science.

steps	Search strategies
#1	TS = (exercise OR physical activity OR sport OR tai chi OR combined exercise OR aerobic exercise OR walking OR pilates OR yoga OR qi gong OR resistance exercise)
#2	TS = (insomnia OR sleep disorder OR sleep complaint OR sleep disturbances OR sleep quality OR sleep problem)
#3	TI = (intervene OR Randomized Controlled Trial OR RCT OR experiment OR trial)
#4	#1 AND #2 AND #3

### 2.2 Inclusion and exclusion criteria

The study strictly formulated the inclusion and exclusion criteria for the selection of literature, adhering to the PICOS principle (Li et al., 2014).

The inclusion criteria for literature consist of the following:

1. Population: There are no age restrictions for the participants included in the study. Additionally, there are no limitations regarding gender or ethnicity.
2. Intervention: The experimental group engaged in long-term exercise, encompassing various types such as aerobic exercise, Traditional Chinese exercises, resistance training, and combined exercise. Moreover, this study imposes no mandatory guidelines regarding the duration of exercise intervention, the intensity of exercise, the exercise environment, nor are there any limitations on the exercise intervention protocol or the approach to blinding.
3. Comparisons: The control group refrains from engaging in any type of exercise intervention and instead continues with their regular daily routines, receiving health education, or undergoing standard care.
4. Outcome: The Pittsburgh Sleep Quality Index (PSQI), a standardized questionnaire comprising 19 items divided into 7 component dimensions, is employed as the metric for measuring sleep quality. It evaluates sleep patterns over the past month, differentiating between individuals with good and poor sleep quality (Bertolazi et al., 2011). Recognized internationally as a reliable sleep quality assessment tool, the PSQI exhibits good internal consistency and test–retest reliability (Hita-Contreras et al., 2014), and has been proven useful in examining sleep quality (Aloba et al., 2007; Mollayeva et al., 2016). Widely applicable and effective (Alsaadi et al., 2013), its validity is widely acknowledged, ensuring high reliability in research and clinical practice (Manzar et al., 2015).
5. Study design: The study includes only Randomized Controlled Trials (RCTs), which ensures the highest level of evidence and minimizes bias (Hannan, 2008), providing a strong guarantee for the reliability of the research results (Lu et al., 2024).

Exclusion criteria were: ① The study methodology excluded non-randomized controlled trials, case reports, animal experimental studies, etc., by categorizing studies based on their type; ② Additionally, literature was classified by type, excluding systematic review articles, qualitative studies, and duplicate publications; ③ Data quality was assessed by excluding literature with unclear measurement indicators or incomplete data, such as studies that do not report PQSI data, or studies that report PSQI data either before or after the intervention.

### 2.3 Data extraction

The study was conducted independently by two researchers, who screened and processed the literature according to predefined criteria, followed by cross-checking. In cases of discrepancies, a third researcher facilitated discussion to reach a consensus before proceeding with data extraction. The extracted data encompassed various key elements, such as the primary author, publication date, country of origin, sample size, age distribution of both experimental and control groups, type of intervention, intervention duration, frequency, exercise duration, intensity, as well as mean, standard deviation, and sample size for sleep quality measurements in both groups.

### 2.4 Basic information extraction

The following is a basic introduction to the data extraction process of this study (for detailed content, see Table 2), all extracted from various studies: participant characteristics (age), exercise characteristics (type of exercise, duration, intervention frequency, period, intensity), and year of publication.

**Table 2:** Explanation of extracted data.

	Definition and comments
Age	The age of the participants, in years, reports their mean age and standard deviation. If the research article clearly distinguishes between the experimental group and the control group, and reports the mean age and standard deviation for both groups, then the age data for the experimental and control groups are clearly labeled in Table 3. If the literature only provides the mean age and standard deviation for the total sample size without further detailing the specific age distribution of the experimental and control groups (including mean and standard deviation), then follow the original data and present it using the mean and standard deviation of the total sample age. For example, in Chen et al.'s (2010) study, since only the overall mean age of the sample (75.40 years) and its standard deviation (6.70 years) were provided, we directly recorded it in Table 3 in the form of “75.40 ± 6.70 “to reflect the data situation of the original literature.
Type	Aerobic exercise, also known as endurance exercise, refers to the type of exercise that the body performs primarily through aerobic metabolism when there is an adequate supply of oxygen (La Touche et al., 2020), characterized by continuity and rhythm, and lower intensity (Senna et al., 2015). Examples include running, walking, yoga (Brown et al., 2022), etc.
	Traditional Chinese fitness exercises refer to fitness systems with Eastern characteristics (Wang et al., 2016). Examples include Tai Chi, Baduanjin, Qigong, martial arts (Yeung et al., 2018), etc.
	Resistance exercise: It refers to a type of exercise where muscles actively contract by overcoming external resistance through their own strength (Stricker et al., 2020). Examples include resistance band training.
	Combined exercise: It refers to a form of exercise that combines two or more of the same or different types of exercises (Oliveira et al., 2012). Examples include aerobic plus resistance training, Tai Chi plus Qigong.
Duration	The duration of time from the start to the end of the intervention, measured in weeks; if the relevant literature presents this period in “months,” it should be converted to “weeks.” This includes durations of ≤4 weeks, 6–8 weeks, 9–10 weeks, 12–16 weeks, and ≥ 24 weeks.
Period	The total duration of each exercise session, including the warm-up, exercise, and cool-down phases, is measured in minutes. This includes durations of ≤30 min, 40–55 min, 60–65 min, and 70 min.
Frequency	The number of exercise sessions per week. This includes 1–2 times, 3 times, 4 times, 5 times, and ≥ 6 times.
Intensity	This study follows the expressions in the original literature when presenting exercise intensity. Given the inconsistency in the standards for presenting exercise intensity in relevant literature, for instance, Luo et al. (2021) used the RPE (Rate of Perceived Exertion) scale, controlling the exercise intensity within the range of RPE = 11–14 (equivalent to low to moderate intensity) (as stated in the original text)(Luo et al., 2021). Alternatively, Ahmad Ali Akbari et al. (2014) used the maximum oxygen uptake to express exercise intensity, setting the low-intensity group at 40–50% MaxHR and the moderate-intensity group at 60–70% MaxHR (Ahmad Ali Akbari et al., 2014). Based on these differences, this study did not redefine or standardize exercise intensity uniformly but instead directly respected the classification terms from the original texts to ensure the accuracy of the representation and the replicability of the study. This includes low intensity, low to moderate intensity, moderate intensity, moderate to high intensity, and high intensity.
Year	Year of publication of the article.

### 2.5 Statistical analysis

Bias risk assessment was conducted using Revman 5.4 software, and network meta-analysis was performed using STATA 17.0 software. Considering the outcome measures as continuous numerical values with significant variations in the measurements across different studies, the Standardized Mean Difference (SMD) was selected, combined with the 95% confidence interval (CI) as the effect size for the synthesis of effect sizes (Chandler et al., 2019). This method effectively pools data from studies of varying sizes (Lu et al., 2024), enhancing the reliability of the conclusions. If the 95% CI of a continuous variable does not include 0, it is considered statistically significant.

When performing network meta-analysis, a network evidence plot should first be drawn to visually display the relationships between direct and indirect comparisons of various exercise interventions. In cases where closed loop structures exist, to ensure the robustness of the results, an inconsistency test is required. If *p* > 0.05, it indicates good consistency between direct and indirect comparisons, meaning the inconsistency test is successful (Salanti et al., 2011). Conversely, if *p* < 0.05, it suggests the presence of inconsistency. Furthermore, when closed loops are present, we also assess the consistency between direct and indirect evidence by calculating the Inconsistency Factors (IF). If the 95% CI of the IF includes 0, it indicates good consistency between direct and indirect evidence, which enhances the reliability of the results.

To more comprehensively assess the robustness of the results, this study employs a leave-one-out method for sensitivity analysis to further verify the reliability of the results. By comparing the surface under the cumulative ranking (SUCRA), the best intervention measures for sleep quality are determined, with a higher SUCRA value indicating a better intervention effect (Daly et al., 2019).

## 3 Results

### 3.1 Literature screening results

**Figure 1:**
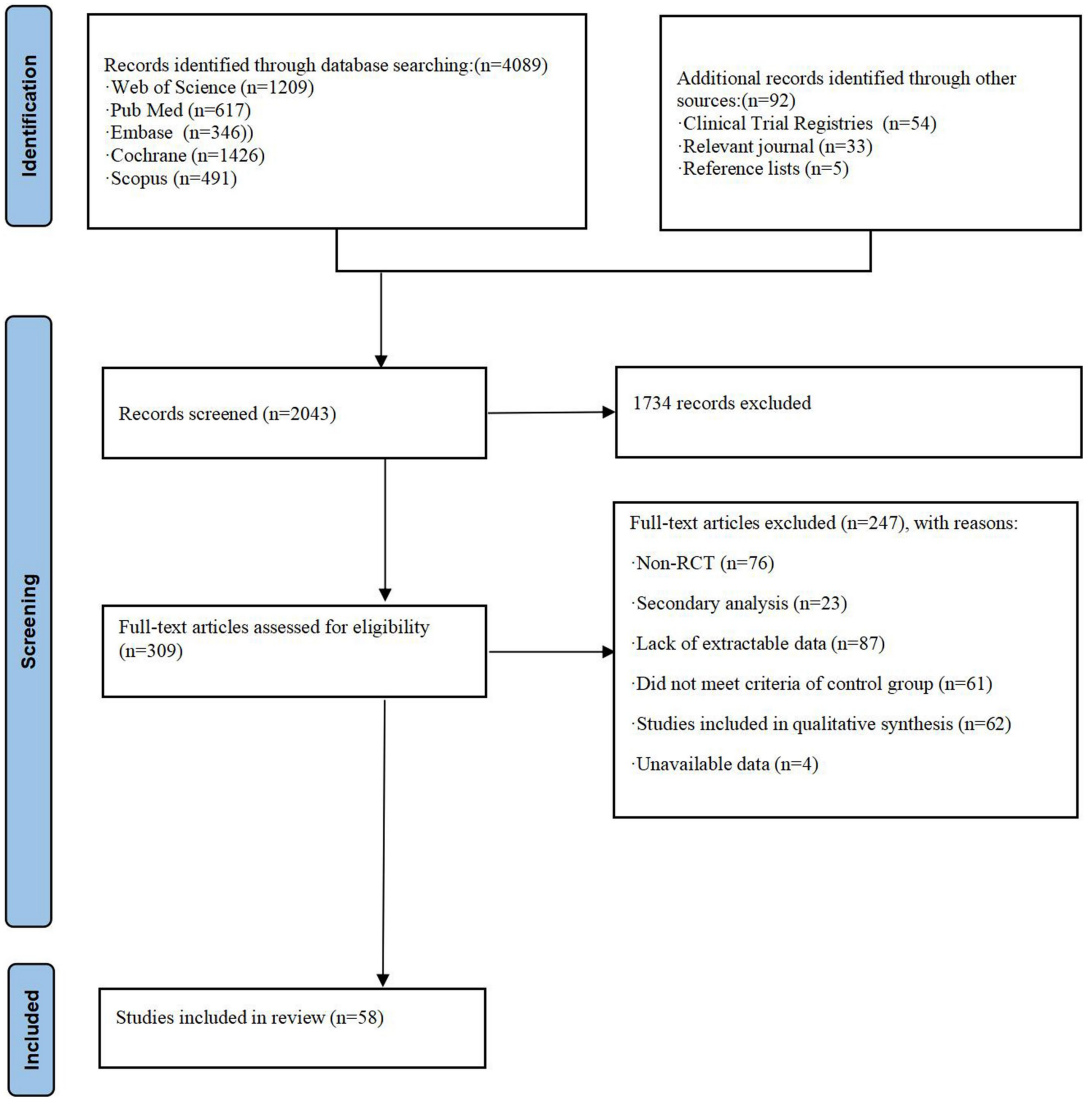
Flow diagram of literature selection process.

The literature screening process was strictly conducted in accordance with the PRISMA flow diagram (Page et al., 2021). A total of 4,181 articles were initially identified through the search process. Following multiple rounds of screening, 58 randomized controlled trials involving 5,008 participants were ultimately included in the analysis. The screening process is illustrated in Figure 1.

### 3.2 Basic characteristics of included studies

Among the 58 studies analyzed, the sample sizes varied from 16 to 393 participants, with ages ranging from 13 to 85 years old. The studies included various types of exercise such as aerobic exercise, traditional Chinese exercises, resistance exercise, and Combined exercise. The intervention periods were conducted at ≤4 weeks, 6–8 weeks, 9–10 weeks, 12–16 weeks, and ≥ 24 weeks, with intervention frequencies of 1–2 times, 3 times, 4 times, 5 times, and ≥ 6 times. The intervention durations were ≤ 30 min, 40–55 min, 60–65 min, and ≥ 70 min. Interventions were conducted at low intensity, low-to-moderate intensity, moderate intensity, moderate-high intensity, and high intensity. All studies are randomized controlled trials (RCTs), and all employ a parallel group design. The basic characteristics of the included literature can be found in Table 3 (When an element represents a range, the average of that range is taken).

**Table 3:** Basic characteristics of included studies.

Author and Year	*N*	Exercise type	Mean age (years)	Country	Dose	Intensity	Research classification	Study design
Ahmad Ali Akbari et al. (2014)	E = 15	Aerobic exercise	60–70	Iran	8 weeks； 2/week； 55 min	Moderate intensity	RCT	Parallel
	E = 15	Aerobic exercise				Low intensity		
	C = 15							
Aibar-Almazán et al. (2019)	E = 55	Aerobic exercise	69.15 ± 8.94	Spain	12 weeks；1/week; 60 min	NR	RCT	Parallel
	C = 52							
Amara et al. (2020)	E = 27	Combined exercise	65.33 ± 8.17	USA	16 weeks; 3/week; 25–55 min	Moderate-high intensity	RCT	Parallel
	C = 28							
Amzajerdi et al. (2023)	E = 40	Aerobic exercise	20.08 ± 1.31	Iran	8 weeks； 3/week； 60 min	NR	RCT	Parallel
	C = 40		20.75 ± 1.27					
Bower et al. (2012)	E = 16	Aerobic exercise	54.4 ± 5.7	USA	12 weeks；2/week； 90 min	NR	RCT	Parallel
	C = 15		53.3 ± 4.9					
Brandão et al. (2018)	E = 64	Combined exercise	68 ± 7	Brazil	12 weeks；3/week 40 min	Moderate intensity	RCT	Parallel
	C = 61							
Chan et al. (2014)	E = 75	Traditional Chinese exercises	39.1 ± 7.9	China	9 weeks；2/week； 90 min	NR	RCT	Parallel
	C = 75		38.9 ± 8.1					
Chen et al. (2009)	E = 62	Aerobic exercise	65.77 ± 4.32	Taiwan, China	24 weeks； 3/week； 70 min	NR	RCT	Parallel
	C = 66		72.42 ± 6.04					
Chen et al. (2010)	E = 31	Aerobic exercise	75.40 ± 6.70	Taiwan, China	24 weeks；3/week; 70 min	NR	RCT	Parallel
	C = 24							
Chen et al. (2012)	E = 27	Traditional Chinese exercises	70.48 ± 7.90	Taiwan, China	12 weeks； 3/week; 30 min	NR	RCT	Parallel
	C = 28		72.96 ± 8.30					
Choi and Sohng (2018)	E = 33	Aerobic exercise	7.6 ± 5.69	South Korea	12 weeks；3/week 30 min	Low intensity	RCT	Parallel
	C = 30							
Courneya et al. (2012)	E = 60	Aerobic exercise	(female)69 ± 59.0	Canada	12 weeks；3/week; 15–45 min	Moderate intensity	RCT	Parallel
	C = 62		(male)48 ± 41.0					
Curi et al. (2018)	E = 31	Aerobic exercise	64.25 ± 0.14	Brazil	16 weeks；2/week; 60 min	NR	RCT	Parallel
	C = 30		63.75 ± 0.08					
del Carmen Carcelén-Fraile et al. (2022)	E = 57	Traditional Chinese exercises	69.73 ± 6.44	Spain	12 weeks；2/week; 60 min	NR	RCT	Parallel
	C = 60							
de Lima et al. (2021)	E = 15	Aerobic exercise	67.2 ± 4.4	Brazil	6 weeks； 3/week; 60 min	NR	RCT	Parallel
	C = 14		68.0 ± 6.1					
Desplan et al. (2014)	E = 11	Combined exercises	30–75	France	4 weeks; 6/week; 120 min	Moderate-high intensity	RCT	Parallel
	C = 11							
Durcan et al. (2014)	E = 40	Resistance exercise	59 ± 12	Ireland	12 weeks; 3/week; 30–60 min	Low-to-mod intensity	RCT	Parallel
	C = 38		61 ± 8					
Eyuboglu et al. (2023)	E = 22	Aerobic exercise	44.36 ± 8.56	Turkey	12 weeks; 3/week; 60 min	NR	RCT	Parallel
	C = 22		42.72 ± 7.57					
Ezati et al. (2020)	E = 32	Aerobic exercise	20.53 ± 1.60	Iran	8 weeks; 3/week; 60 min	Low-to-mod intensity	RCT	Parallel
	C = 35		20.08 ± 1.31					
Fan et al. (2020)	E = 67	Traditional Chinese exercises	71.1 ± 6.3	China	24 weeks; 5/week; 45 min	NR	RCT	Parallel
	C = 72							
Frye et al. (2007)	E1 = 23	Traditional Chinese exercises	69.2 ± 9.26	USA	12 weeks; 5/week; 60 min	Low intensity	RCT	Parallel
	E2 = 28	Aerobic exercise			12 weeks; 3/week; 60 min	NR		
	C = 21							
Ghaffarilaleh et al. (2019)	E = 26	Aerobic exercise	34.46 ± 5.37	Iran	10 weeks; 3/week; 20 min	NR	RCT	Parallel
	C = 28		30.18 ± 6.29					
Ghavami and Akyolcu (2017)	E = 40	Aerobic exercise	48.75 ± 9.49	Turkey	24 weeks; 3–5/week; 50 min	Moderate intensity	RCT	Parallel
	C = 40		49.23 ± 9.46					
Hariprasad et al. (2013)	E = 44	Aerobic exercise	75.74 ± 6.46	India	4 weeks; 7/week; 60 min	NR	RCT	Parallel
	C = 43		74.78 ± 7.35					
Hosseini et al. (2011)	E = 31	Traditional Chinese exercises	68.74 ± 5.48	Iran	12 weeks; 3/week; 40 min	NR	RCT	Parallel
	C = 31		69.42 ± 5.34					
Huang et al. (2023)	E = 30	Traditional Chinese exercises	36.85 ± 8.72	China	12 weeks; 4/week; 60 min	NR	RCT	Parallel
	E = 30	Aerobic exercise						
	C = 29							
Irwin et al. (2008)	E1 = 29	Traditional Chinese exercises	69.6 ± 6.3	USA	25 weeks; 3/week; 40 min	NR	RCT	Parallel
	C = 31		69.9 ± 7.6					
	E2 = 30	Traditional Chinese exercises	69.7 ± 6.1		16 weeks; 3/week; 40 min			
	C = 22		70.7 ± 7.5					
Irwin et al. (2014)	E = 48	Traditional Chinese exercises	66.3 ± 7.4	USA	16 weeks; 120 min/week	NR	RCT	Parallel
	C = 25		66.4 ± 7.7					
Irwin et al. (2017)	E = 33	Traditional Chinese exercises	59.6 ± 7.9	USA	3 weeks; 3/week; 40 min	NR	RCT	Parallel
	C = 40		60.0 ± 9.3					
Jiménez-García et al. (2021)	E1 = 26	Resistance exercise	68.23 ± 2.77	Spain	12 weeks; 2/week; 45 min	High intensity	RCT	Parallel
	E2 = 24	Resistance exercise	68.75 ± 5.98			Moderate intensity		
	C = 23		68.52 ± 6.33					
Karimi et al. (2016)	E = 23	Aerobic exercise	67.49 ± 4.28	Iran	8 weeks; 3/week; 30 min	NR	RCT	Parallel
	C = 23		66.82 ± 3.84					
King et al. (2008)	E = 36	Aerobic exercise	61.86 ± 6.33	USA	48 weeks; 5/week; 60 min	Moderate intensity	RCT	Parallel
	C = 30		60.90 ± 7.19					
Kline et al. (2011)	E = 21	Combined exercise	47.6 ± 1.3	USA	12 weeks; 4/week; 50 min	Moderate intensity	RCT	Parallel
	C = 16		45.9 ± 2.2					
Li et al. (2004)	E = 62	Traditional Chinese exercises	75.30 ± 7.8	USA	24 weeks; 2/week; 60 min	NR	RCT	Parallel
	C = 56		75.45 ± 7.8					
Luo et al. (2021)	E = 23	Aerobic exercise	13.83 ± 0.39	China	12 weeks; 4/week; 30 min	Low-to-mod intensity	RCT	Parallel
	C = 34		13.96 ± 0.21					
McKenna et al. (2021)	E = 10	Aerobic exercise	57 ± 7.5	Ireland	9 weeks 5/week 30 min	Moderate intensity	RCT	Parallel
	C = 10							
Mercier et al. (2018)	E = 20	Aerobic exercise	56.6 ± 10.2	Canada	6 weeks; 7/week; 90 min	NR	RCT	Parallel
	C = 21		57.6 ± 9.3					
Mustian et al. (2013)	E = 197	Aerobic exercise	54.1 ± 0.51	USA	4 weeks; 2/week; 75 min	NR	RCT	Parallel
	C = 196							
Ng et al. (2022)	E = 95	Traditional Chinese exercises	55.97 ± 10.76	Hong Kong, China	8 weeks; 3/week; 60 min	NR	RCT	Parallel
	C = 93							
Nguyen and Kruse (2012)	E = 39	Traditional Chinese exercises	69.2 ± 5.3	Vietnam	24 weeks; 2/week; 60 min	NR	RCT	Parallel
	C = 34		68.7 ± 4.9					
Serrano-Guzman et al. (2016)	E = 35	Aerobic exercise	69.07 ± 4.4	Spain	8 weeks; 3/week; 50 min	Low intensity	RCT	Parallel
	C = 32		69.48 ± 3.2					
Shen and Chen (2021)	E = 61	Aerobic exercise	33.3	Taiwan, China	12 weeks; 3/week; 20 min	Low intensity	RCT	Parallel
	C = 62		32.8					
Singh et al. (1997)	E = 15	Resistance training	70.0 ± 1.6	USA	10 weeks; 3/week; 65 min	High intensity	RCT	Parallel
	C = 13		72.0 ± 1.9					
Siu et al. (2021)	E1 = 93	Traditional Chinese exercises	66.5 ± 6.4	Hong Kong, China	12 weeks; 3/week 60 min	NR	RCT	Parallel
	E2 = 98	Aerobic exercise	67.3 ± 5.7					
	C = 94		68.0 ± 8.2					
Song et al. (2022)	E = 28	Traditional Chinese exercises	64.15 ± 8.56	Hong Kong, China	12 weeks; 3/week; 60 min	NR	RCT	Parallel
	C = 20							
Song et al. (2024)	E = 45	Aerobic exercise	75.97 ± 6.31	Hong Kong, China	16 weeks; 3/week; 60 min	Moderate intensity	RCT	Parallel
	C = 44							
Souza et al. (2018)	E = 8	Aerobic exercise	54.8 ± 6.9	Brazil	12 weeks; 2/week; 30 min	Moderate intensity	RCT	Parallel
	C = 8		49.9 ± 11.6					
Suna et al. (2015)	E = 54	Combined exercise	61 ± 15	Australia	12 weeks; 2/week; 60 min	Low-to-mod intensity	RCT	Parallel
	C = 52		62 ± 13					
Tang et al. (2010)	E = 24	Aerobic exercise	47.36 ± 10.14	Taiwan, China	8 weeks; 3/week; 40 min	Low-to-mod intensity	RCT	Parallel
	C = 35		56.3 ± 12.43					
Taylor-Piliae and Coull (2012)	E = 13	Traditional Chinese exercises	72.8 ± 10.1	USA	12 weeks; 3/week; 60 min	NR	RCT	Parallel
	C = 12		64.5 ± 10.9					
Tseng et al. (2020)	E = 20	Aerobic exercise	61.1 ± 6.8	Taiwan, China	12 weeks; 3/week; 50 min	Moderate intensity	RCT	Parallel
	C = 20		62.2 ± 7.4					
Viswanathan et al. (2021)	E = 150	Aerobic exercise	50.8 ± 8.3	India	12 weeks; 5/week; 50 min	NR	RCT	Parallel
	C = 150		52.8 ± 7.0					
Wang et al. (2010)	E = 17	Traditional Chinese exercises	76.53 ± 9.74	Japan	12 weeks; 1/week; 50 min	NR	RCT	Parallel
	C = 17		77.59 ± 12.33					
Wenzel et al. (2013)	E = 68	Aerobic exercise	60.2 ± 10.6	USA	5–35 weeks; 3/week; 60 min	Moderate intensity	RCT	Parallel
	C = 58							
Yeh and Chang (2012)	E = 35	Traditional Chinese exercises	48.60 ± 1.94	Taiwan, China	12 weeks; 7/week; 30 min	NR	RCT	Parallel
	C = 35		48.69 ± 2.04					
Yilmaz Gokmen et al. (2019)	E = 25	Combined exercise	50.44 ± 8.38	Turkey	12 weeks；3/week; 60 min	Low-to-mod intensity	RCT	Parallel
	C = 25							
Zeibig et al. (2021)	E = 36	Aerobic exercise	37.33 ± 14.23	Germany	12 weeks; 4/week; 30 min	Moderate intensity	RCT	Parallel
	C = 36		34.33 ± 12.39					
Zhou et al. (2022)	E = 15	Aerobic exercise	79.46 ± 4.82	China	12 weeks; 3/week; 60 min	Moderate intensity	RCT	Parallel
	E = 17	Resistance exercise	78.88 ± 5.40			Moderate intensity		
	E = 17	Combined exercise	78.88 ± 5.40			Moderate intensity		
	E = 17	Combined exercise	78.41 ± 6.12			Moderate intensity		
	C = 17		75.41 ± 19.35					

E, Experimental Group; C, Control Group; NR, Not Reported. All outcome measures are PSQI. Age is presented as the mean ± standard deviation.

### 3.3 Traditional meta-analysis

**Figure 2:**
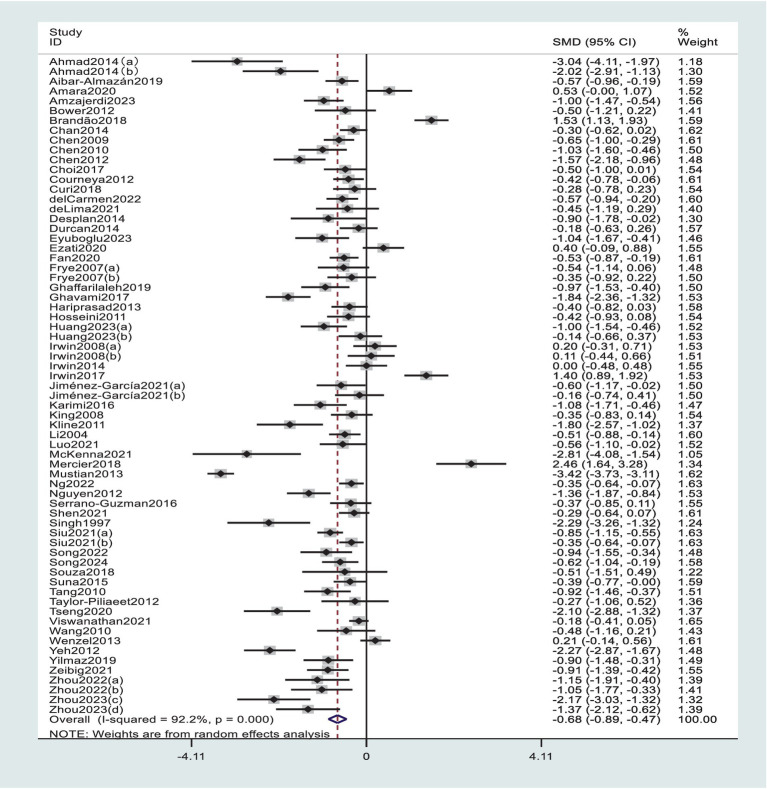
Forest plot of the effect of exercise intervention on sleep quality.

Due to the presence of high heterogeneity, a random-effects model was used for the meta-analysis, and the results are depicted in Figure 2. The pooled effect size SMD is −0.68 (*p* < 0.0001), with a 95% confidence interval of (−0.89, −0.47). This indicates that exercise intervention has a significant promoting effect on the improvement of sleep quality. *p* < 0.0001 indicates that the pooled effect size from multiple studies is statistically significant, thus suggesting that compared to the control group, exercise intervention can effectively enhance sleep quality.

### 3.4 Risk of bias assessment

**Figure 3:**
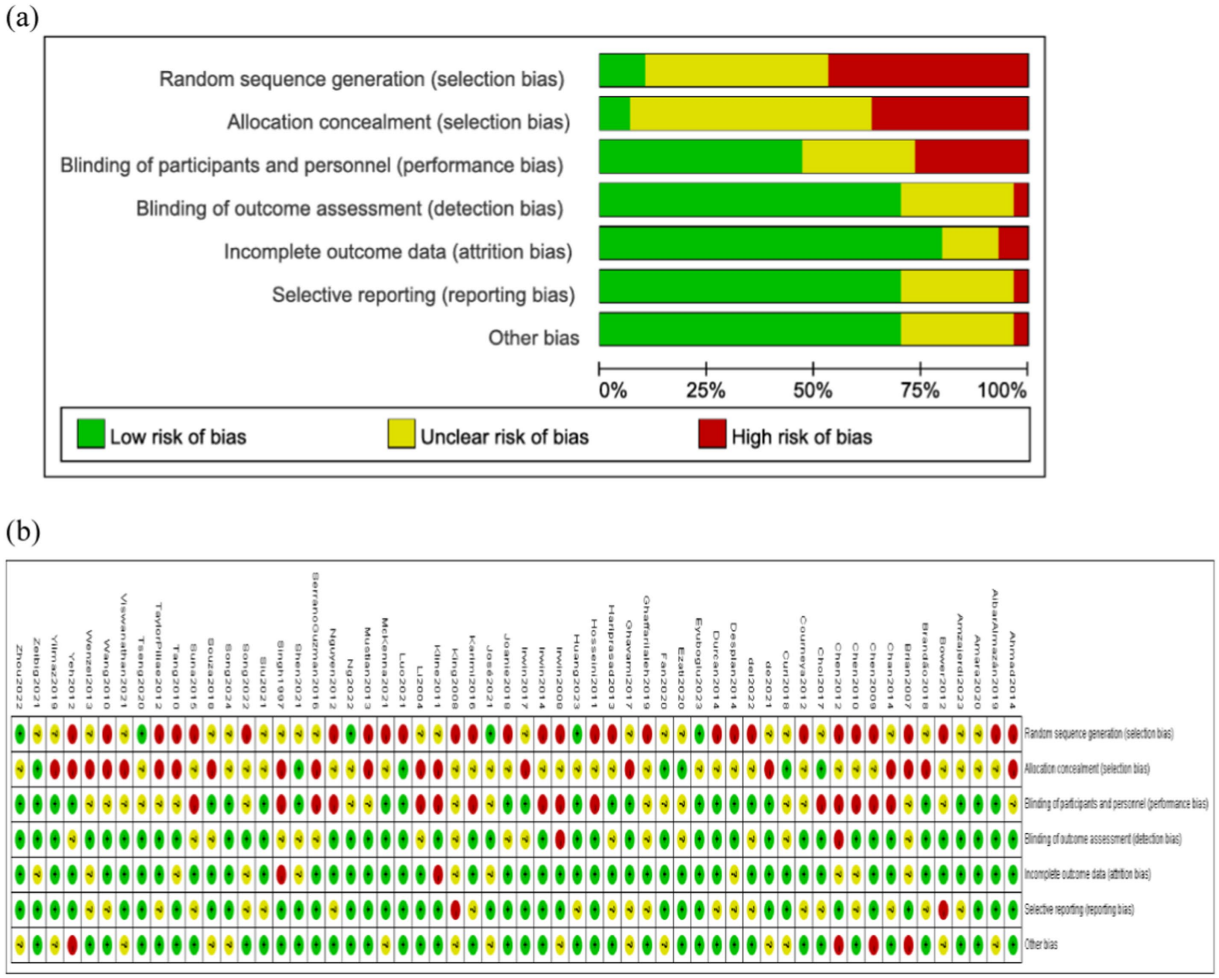
Bias risk assessment of included studies. **(a)** Risk of bias summary. **(b)** Risk of bias graph.

The quality assessment of the studies was conducted using the Cochrane Risk of Bias tool (Higgins, 2011), which evaluates seven aspects: Random Sequence Generation, Allocation Concealment, Blinding of Participants and Personnel, Blinding of Outcome Assessment, Incomplete Outcome Data, Selective Reporting, and Other Bias. Each item in the assessment offers three options: “Low risk of bias,” “Unclear risk of bias,” and “High risk of bias” (Jadad et al., 1996). This assessment was carried out independently by two researchers, with a third researcher involved in resolving any discrepancies through discussion to reach a consensus. The risk of bias is visually represented in Figure 3.

### 3.5 Sensitivity analyses

From Table 4, it can be seen that after the examination using the one-by-one exclusion method, the change in the effect size is minimal. This indicates that the removal of any single document did not significantly affect the overall combined effect size, suggesting that the analysis results of this study are robust, and the combined effect size is minimally influenced by any individual document.

**Table 4:** Sensitivity analysis of sleep quality in exercise intervention.

Author and Year	Estimate	95%CI	Author and Year	Estimate	95%CI
Ahmad Ali Akbari et al. (2014)	−0.65	−0.86, −0.44	Jiménez-García et al. (2021)	−0.69	−0.90, −0.47
Ahmad Ali Akbari et al. (2014)	−0.66	−0.88, −0.45	Karimi et al. (2016)	−0.67	−0.89, −0.46
Aibar-Almazán et al. (2019)	−0.68	−0.90, −0.47	King et al. (2008)	−0.69	−0.90, −0.47
Amara et al. (2020)	−0.70	−0.91, −0.49	Kline et al. (2011)	−0.66	−0.88, −0.45
Amzajerdi et al., 2023	−0.68	−0.89, −0.46	Li et al. (2004)	−0.68	−0.90, −0.47
Bower et al. (2012)	−0.68	−0.90, −0.47	Luo et al. (2021)	−0.68	−0.90, −0.47
Brandão et al. (2018)	−0.71	−0.91,0.51	McKenna et al. (2021)	−0.66	−0.87, −0.45
Chan et al. (2014)	−0.69	−0.90, −0.47	Mercier et al. (2018)	−0.72	−0.93, −0.51
Chen et al. (2009)	−0.68	−0.90, −0.47	Mustian et al. (2013)	−0.62	−0.79, −0.45
Chen et al., 2010	−0.68	−0.89, −0.46	Ng et al. (2022)	−0.69	−0.90, −0.47
Chen et al. (2012)	−0.67	−0.88, −0.45	Nguyen and Kruse (2012)	−0.67	−0.88, −0.46
Choi (2018)	−0.68	−0.90, −0.47	Serrano-Guzman et al. (2016)	−0.69	−0.90, −0.47
Courneya et al. (2012)	−0.69	−0.90, −0.47	Shen and Chen (2021)	−0.69	−0.90, −0.47
Curi et al. (2018)	−0.69	−0.90, −0.47	Singh et al. (1997)	−0.66	−0.87, −0.45
del Carmen Carcelén-Fraile et al. (2022)	−0.68	−0.90, −0.47	Siu et al. (2021)	−0.68	−0.90, −0.46
de Lima et al., 2021	−0.68	−0.90, −0.47	Siu et al. (2021)	−0.69	−0.90, −0.47
Desplan et al. (2014)	−0.68	−0.89, −0.46	Song et al. (2022)	−0.68	−0.89, −0.46
Durcan et al. (2014)	−0.69	−0.90, −0.47	Song et al. (2024)	−0.68	−0.90, −0.47
Eyuboglu et al. (2023)	−0.68	−0.89, −0.46	Souza et al. (2018)	−0.68	−0.90, −0.47
Ezati et al. (2020)	−0.70	−0.91, −0.48	Suna et al. (2015)	−0.69	−0.90, −0.47
Fan et al. (2020)	−0.68	−0.90, −0.47	Tang et al. (2010)	−0.68	−0.89, −0.46
Frye et al. (2007)	−0.68	−0.90, −0.47	Taylor-Piliae and Coull (2012)	−0.69	−0.90, −0.47
Frye et al. (2007)	−0.69	−0.90, −0.47	Tseng et al. (2020)	−0.66	−0.87, −0.45
Ghaffarilaleh et al. (2019)	−0.68	−0.89, −0.46	Viswanathan et al. (2021)	−0.69	−0.91, −0.47
Ghavami and Akyolcu (2017)	−0.66	−0.87, −0.45	Wang et al. (2010)	−0.68	−0.90, −0.47
Hariprasad et al. (2013)	−0.69	−0.90, −0.47	Wenzel et al. (2013)	−0.70	−0.91, −0.48
Hosseini et al. (2011)	−0.68	−0.90, −0.47	Yeh and Chang (2012)	−0.66	−0.87, −0.45
Huang et al., 2023	−0.68	−0.89, −0.46	Yilmaz Gokmen et al. (2019)	−0.68	−0.89, −0.46
Huang et al., 2023	−0.69	−0.90, −0.48	Zeibig et al. (2021)	−0.68	−0.89, −0.46
Irwin et al. (2008)	−0.69	−0.91, −0.48	Zhou et al. (2022)	−0.67	−0.89, −0.46
Irwin et al. (2008)	−0.69	−0.91, −0.48	Zhou et al. (2022)	−0.68	−0.89, −0.46
Irwin et al. (2014)	−0.69	−0.91, −0.48	Zhou et al. (2022)	−0.66	−0.87, −0.45
Irwin et al. (2017)	−0.71	−0.92, −0.50	Zhou et al. (2022)	−0.67	−0.88, −0.46
Jiménez-García et al. (2021)	−0.68	−0.90, −0.47	Combined	−0.68	−0.89, −0.47

### 3.6 Consistency tests

Network meta-analysis relies on the assumption of consistency, where consistency tests evaluate the coherence between specific direct and indirect evidence (defined as comparisons) (Dias et al., 2013). During the process of conducting a network meta-analysis, when a closed loop structure exists among various interventions, consistency testing is required to assess the degree of agreement between the results of direct and indirect comparisons (White et al., 2012). The *p*-value for the global consistency test of exercise type was 0.59, exceeding the significance threshold of 0.05, suggesting satisfactory overall consistency. Further examination of the consistency within each closed loop of exercise type revealed 95% confidence intervals (CI) of [0.00,3.60], [0.00,2.86], [0.00,2.99], and [0.00,1.76], with all lower bounds of the inconsistency factors including 0, suggesting good loop consistency. The global consistency test for exercise intensity (Intensity) resulted in a p-value of 0.065, which is also greater than 0.05, indicating good global consistency. Further examination of the consistency within each closed loop of exercise intensity showed 95% CI of [0.00,2.55] and [0.00,2.76], with both lower bounds of the inconsistency factors including 0, indicating good loop consistency. Consequently, a consistency model was adopted for analysis. Exercise frequency, exercise duration, and exercise period did not form closed loops, hence no consistency tests were required.

### 3.7 Network meta-analysis results

#### 3.7.1 Network plot

**Figure 4:**
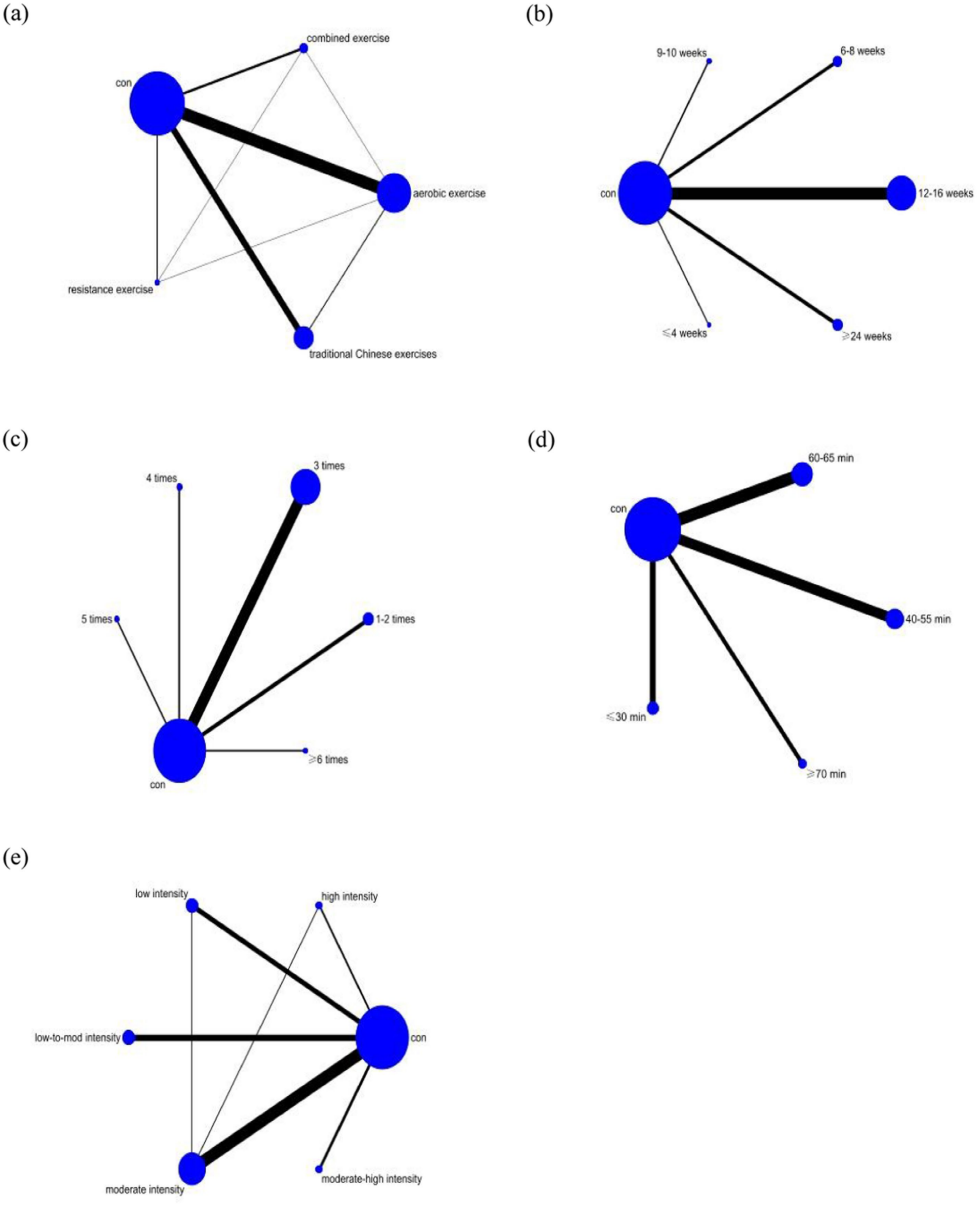
Network meta-analysis intervention diagram. **(a)** Type of exercise. **(b)** Exercise duration. **(c)** Exercise frequency. **(d)** Duration of a single exercise session. **(e)** Exercise intensity. Con, control group.

In the network plot, the size of the circles corresponds to the sample size for each intervention, with larger circles indicating a greater number of samples. The lines connecting the circles represent comparisons between different interventions, and the thickness of the lines reflects the number of studies included in each comparison. The specific network plot is depicted in Figure 4 (The average number of elements is considered for elements that fall within a certain range).

#### 3.7.2 Pairwise meta-analysis results

As shown in Table 5, through pairwise comparisons, it was found that the intervention effects of aerobic exercise [SMD = −0.56, 95% CI (−0.86, −0.27)], traditional Chinese exercises [SMD = −0.57, 95% CI (−0.97, −0.18)], and combined exercise [SMD = −0.99, 95% CI (−1.66, −0.32)] were significantly better than those of the control group. This indicates that, compared to the control group, aerobic exercise, traditional Chinese exercises, and combined exercise are more effective in promoting sleep quality. The intervention periods of 9–10 weeks [SMD = −1.40, 95% CI (−2.37, −0.44)], 12–16 weeks [SMD = −0.55, 95% CI (−0.90, −0.20)], and ≥ 24 weeks [SMD = −0.71, 95% CI (−1.31, −0.10)] were significantly better than the control group, and the intervention effect of 9–10 weeks was significantly better than that of 6–8 weeks [SMD = −1.21, 95% CI (−2.37, −0.04)]. This suggests that, compared to the control group, intervention periods of 9–10 weeks, 12–16 weeks, and ≥ 24 weeks are more effective in promoting sleep, and the 9–10 weeks period is more conducive to improving sleep quality than the 6–8 weeks period. The intervention frequency of 1–2 times per week [SMD = −0.85, 95% CI (−1.43, −0.26)], 3 times per week [SMD = −0.45, 95% CI (−0.80, −0.11)], and 4 times per week [SMD = −1.09, 95% CI (−1.92, −0.26)] was significantly better than the control group. This indicates that, compared to the control group, intervention frequencies of 1–2 times, 3 times, and 4 times per week are more effective in promoting sleep. The intervention duration of ≤30 min and 60–65 min was significantly better than the control group, and the intervention effect of ≤30 min was significantly better than that of 40–55 min [SMD = 0.75, 95% CI (−1.49, −0.01)]. This suggests that, compared to the control group, interventions of ≤30 min and 60–65 min are more effective in promoting sleep, and the intervention of ≤30 min is more conducive to improving sleep quality than the 40–55 min intervention. The intervention intensity of moderate intensity [SMD = −1.06, 95% CI (−1.52, −0.61)] and high intensity [SMD = −1.48, 95% CI (−2.55, −0.40)] was significantly better than the control group, and both high intensity [SMD = −1.97, 95% CI (−3.37, −0.56)] and moderate intensity [SMD = −1.55, 95% CI (−2.57, −0.54)] interventions were significantly better than moderate-high intensity. This indicates that, compared to the control group, interventions of moderate and high intensity are more effective in promoting sleep, and interventions of moderate and high intensity are more conducive to improving sleep quality than moderate-high intensity interventions.

**Table 5:** League table of intervention effects for various exercise prescription elements.

Combined exercise					
−0.17 (−1.31,0.96)	Resistance exercise				
−0.42 (−1.19,0.36)	−0.24 (−1.30,0.81)	Traditional Chinese exercises			
−0.42 (−1.15,0.30)	−0.25 (−1.26,0.76)	−0.01 (−0.48,0.47)	Aerobic exercise		
**−0.99 (−1.66, −0.32)**	−0.81 (−1.80,0.17)	**−0.57 (−0.97, −0.18)**	**−0.56 (−0.86, −0.27)**	con	
≥24 weeks					
−0.16 (−0.85,0.54)	12–16 weeks				
0.70 (−0.44,1.83)	0.85 (−0.17,1.88)	9–10 weeks			
−0.51 (−1.40,0.38)	−0.35 (−1.09,0.38)	**−1.21 (−2.37, −0.04)**	6–8 weeks		
0.31 (−0.92,1.53)	0.46 (−0.66,1.59)	−0.39 (−1.83,1.05)	0.82 (−0.43,2.07)	≤4 weeks	
**−0.71 (−1.31, −0.10)**	**−0.55 (−0.90, −0.20)**	**−1.40 (−2.37, −0.44)**	−0.19 (−0.84,0.46)	−1.01 (−2.08,0.05)	con
≥6 times					
0.47 (−0.87,1.81)	5 times				
0.78 (−0.49,2.04)	0.31 (−0.95,1.57)	4 times			
0.14 (−0.87,1.15)	−0.33 (−1.33,0.67)	−0.64 (−1.54,0.26)	3 times		
0.53 (−0.59,1.65)	0.06 (−1.05,1.17)	−0.25 (−1.26,0.77)	0.39 (−0.29,1.07)	1–2 times	
−0.31 (−1.27,0.64)	−0.78 (−1.73,0.16)	**−1.09 (−1.92, −0.26)**	**−0.45 (−0.80, −0.11)**	**−0.85 (−1.43, −0.26)**	con
≥70 min					
−0.09 (−0.90,0.72)	60–65 min				
−0.36 (−1.19,0.47)	−0.27 (−0.89,0.35)	40–55 min			
0.39 (−0.52,1.30)	0.48 (−0.24,1.20)	**0.75 (0.01,1.49)**	≤30 min		
−0.68 (−1.37,0.01)	**−0.59 (−1.00, −0.17)**	−0.32 (−0.77,0.14)	**−1.07 (−1.65, −0.48)**	con	
High intensity					
**−1.97 (−3.37, −0.56)**	Moderate-high intensity				
−0.41 (−1.52,0.69)	**1.55 (0.54,2.57)**	Moderate intensity			
−1.06 (−2.31,0.18)	0.90 (−0.20,2.00)	−0.65 (−1.42,0.12)	Low-to-mod intensity		
−0.96 (−2.22,0.30)	1.01 (−0.12,2.14)	−0.54 (−1.32,0.24)	0.11 (−0.81,1.02)	Low intensity	
**−1.48 (−2.55, −0.40)**	0.49 (−0.42,1.40)	**−1.06 (−1.52, −0.61)**	−0.41 (−1.04,0.21)	−0.52 (−1.19,0.15)	con

Numbers in bold indicate statistical significance. Con, control group.

#### 3.7.3 Ranking of the effectiveness of each exercise prescription element

**Figure 5:**
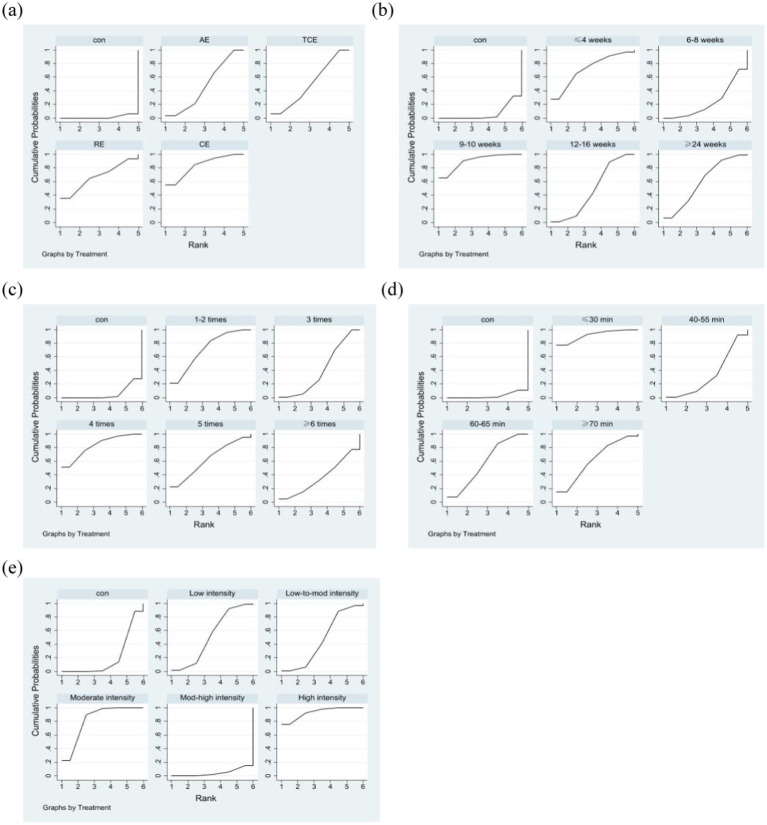
Probability ranking plot of the intervention effects of various exercise prescription elements. **(a)** Type of exercise. **(b)** Exercise duration. **(c)** Exercise frequency. **(d)** Duration of a single exercise session. **(e)** Exercise intensity. Con, control group.

According to the SUCRA method, the ranking of the impact of various types of exercise on sleep quality, as illustrated in Table 6, is as follows: combined exercise (SUCRA = 82.7) > resistance exercise (SUCRA = 67.2) > traditional Chinese exercises (SUCRA = 50.1) > aerobic exercise (SUCRA = 48.8). The ranking of the impact of different frequencies of exercise interventions on sleep quality is as follows: 4 times per week (SUCRA = 84.7) > 1–2 times per week (SUCRA = 70.6) > 5 times per week (SUCRA = 64.4) > 3 times per week (SUCRA = 41.2) > ≥6 times per week (SUCRA = 32.4). The ranking of the impact of various intensities of exercise interventions on sleep quality is as follows: high intensity (SUCRA = 92.9) > moderate intensity (SUCRA = 81.8) > low intensity (SUCRA = 53.0) > low-to-mod intensity (SUCRA = 47.6) > moderate-high intensity (SUCRA = 4.2). The ranking of the impact of various exercise durations on sleep quality is as follows: 9–10 weeks (SUCRA = 89.9) > ≤4 weeks (SUCRA = 72.7) > ≥24 weeks (SUCRA = 60.8) > 12–16 weeks (SUCRA = 48.2) > 6–8 weeks (SUCRA = 22.8). The ranking of the impact of different single intervention durations on sleep quality is as follows: ≤30 min (SUCRA = 92.2) > ≥ 70 min (SUCRA = 62.7) > 60–65 min (SUCRA = 58.7) > 40–55 min (SUCRA = 33.7) (Figure 5).

**Table 6:** SUCRA values for the effectiveness of each exercise prescription element.

Period	SUCRA	Frequency	SUCRA	Intensity	SUCRA	Type	SUCRA	Duration	SUCRA
Con	5.6	Con	6.6	Con	20.6	Con	1.3	con	2.7
≤4 weeks	72.7	1–2 times	70.6	Low intensity	53.0	Aerobic exercise	48.8	≤30 min	92.2
6–8 weeks	22.8	3 times	41.2	Low-to-mod intensity	47.6	Traditional Chinese exercises	50.1	40–55 min	33.7
9–10 weeks	89.9	4 times	84.7	Moderate intensity	81.8	Resistance exercise	67.2	60–65 min	58.7
12–16 weeks	48.2	5 times	64.4	Moderate-high intensity	4.2	Combined exercise	82.7	≥70 min	62.7
≥24 weeks	60.8	≥6 times	32.4	High intensity	92.9				

Con, control group.

#### 3.7.4 Publication bias test

**Figure 6:**
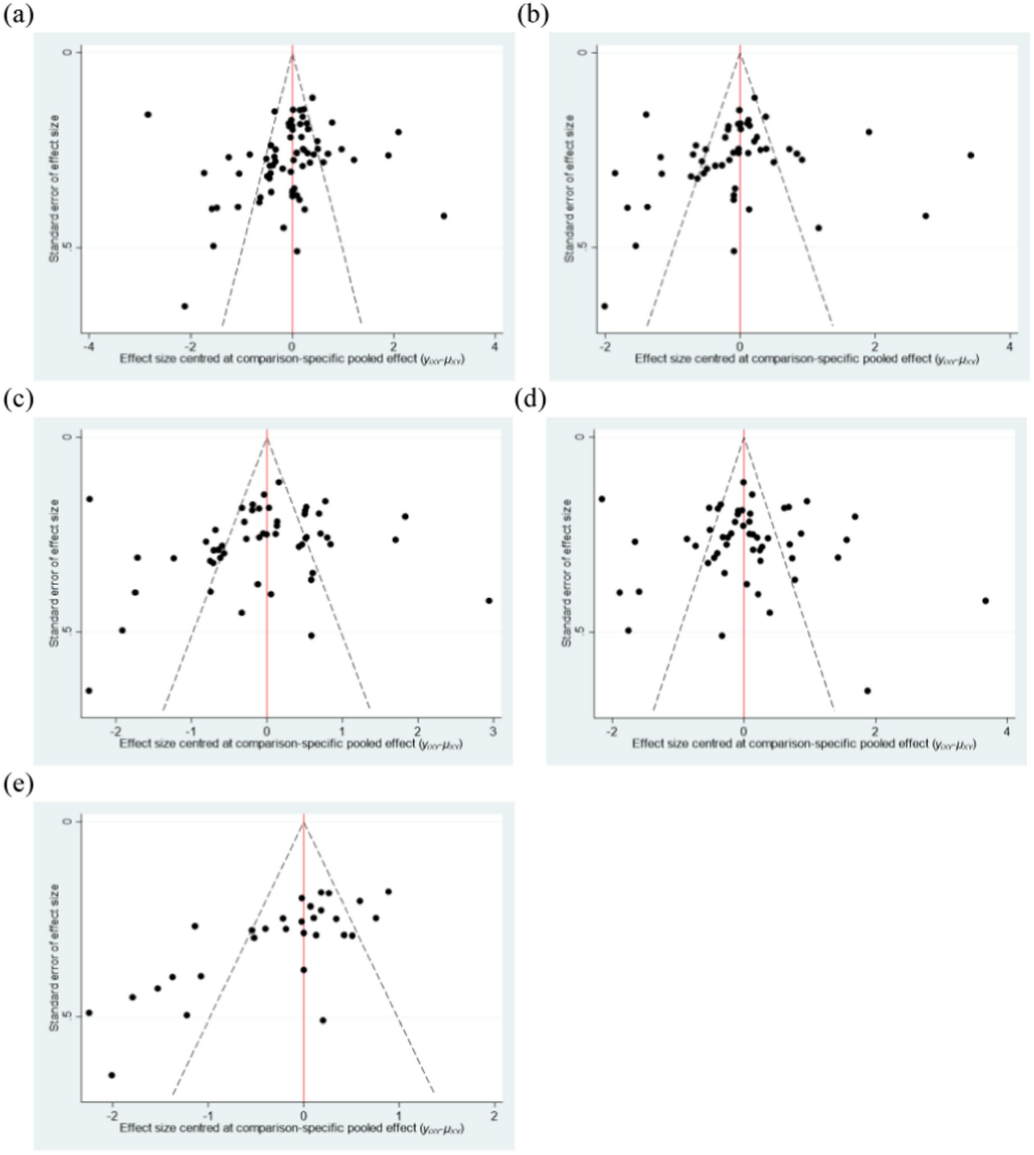
Funnel plot of the intervention effects of various elements of the exercise program. **(a)** Type of exercise. **(b)** Exercise duration. **(c)** Exercise frequency. **(d)** Duration of a single exercise session. **(e)** Exercise intensity. Con, control group.

To examine the potential publication bias in network meta-analysis that may be caused by small-scale studies, funnel plots are used for analysis. Funnel plots can be visually inspected for symmetry, which effectively helps us determine whether there is bias caused by small-scale studies, providing an important basis for the reliability of the research results (Khera et al., 2016). From Figure 6, it can be observed that the distribution of study points on both sides of the inverted funnel plot is relatively regular, with only a few study points scattered, suggesting that the included literature has a low likelihood of publication bias.

Furthermore, this study also conducted a test for publication bias using the Egger method. The *p*-value was 0.167, with a 95% CI (−4.417, 0.777). This indicates that there is no significant publication bias.

## 4 Discussion

This study compares the effects of different interventions on sleep quality through a network meta-analysis of existing research. We conducted a comprehensive and in-depth synthesis analysis, including direct and indirect evidence from 58 randomized controlled trials involving 5,008 participants who underwent various exercise programs to analyze their impact on sleep quality. The purpose of this study is to address the limitations of previous meta-analyses that were often confined to a single aspect or immediate exercise effects by exploring the impact of exercise frequency, duration, period, intensity, and type on multiple dimensions of sleep quality.

In recent years, exercise has been widely recognized for its role in improving sleep quality. Specifically, aerobic exercise, traditional Chinese exercises, resistance exercise, and combined exercise have shown unique therapeutic effects, each promoting the enhancement of sleep quality through different mechanisms. Aerobic exercise can increase aerobic capacity parameters, reduce REM latency, and decrease wakefulness (Santos et al., 2012). Traditional Chinese exercises, such as Qigong and Tai Chi, significantly enhance the tone of the vagus nerve by slowing down bodily movement and respiratory frequency, thereby promoting the balance of the autonomic nervous system (ANS). This physiological regulatory process not only adjusts the reactivity of the hypothalamic–pituitary–adrenal axis (HPA axis) but also leads to a significant reduction in serum cortisol levels. This effectively alleviates stress responses and significantly improves sleep quality (Mudano et al., 2019). Resistance exercise is an enhancer of brain-derived neurotrophic factor (BDNF), with increased BDNF concentration levels in the brain after exercise (Babaei et al., 2013), promoting the connection and reorganization of neurons in the brain, further optimizing neural networks related to sleep regulation, and thus facilitating sleep initiation and maintenance (Chennaoui et al., 2015). Combined exercise has been found to be more effective than single exercise modalities in improving sleep quality (Zhou et al., 2022). The network meta-analysis concluded that: a frequency of exercise four times per week is more effective in improving sleep quality; a single exercise session duration of ≤30 min is more effective in improving sleep quality; a 9–10 weeks exercise intervention period is more effective in improving sleep quality; high-intensity exercise is more effective in improving sleep quality; and combined exercise is more effective in improving sleep quality.

This study found that combined exercise is the best type of exercise to improve sleep quality, which is inconsistent with some existing research results. Previous meta-analysis results have shown that aerobic exercise has the most significant effect on improving sleep quality (Fang et al., 2019). The reason may be that combined exercise can leverage the advantages of various types of exercise. Research has indicated that a combined program of aerobic and resistance exercise demonstrates superior effects in improving sleep quality (Brellenthin and Lee, 2022). This is due to the fact that aerobic exercise has been shown to positively impact the release of serotonin and noradrenaline, essential neurotransmitters for enhancing sleep quality (Dolezal et al., 2017). Additionally, aerobic exercise can increase melatonin levels, which aid in improving sleep propensity (Kline et al., 2011), regulating the sleep–wake cycle, and facilitating better and faster sleep at night (Chen et al., 2024). On the other hand, resistance exercise has been found to reduce sleep onset latency, promote faster sleep onset, decrease the number of awakenings after sleep onset, and improve sleep efficiency (Zhou et al., 2022). Consequently, the combined exercises demonstrate enhanced effects on improving sleep quality.

A frequency of exercise four times per week is most effective for improving sleep quality, a finding that is consistent with previous research conclusions (Feitl et al., 2009). Ezati’s investigation proposes that exercise regimens consisting of four to seven sessions weekly are linked to improved sleep quality (Ezati et al., 2020), while other studies have demonstrated that exercising at least three times per week yields more significant enhancements in sleep quality (Hasan et al., 2022). This phenomenon may be attributed to the modulatory impact of exercise frequency on mood, as evidenced by studies indicating that regular exercise at a frequency of four times per week can enhance the release of endorphins (Huang et al., 2023). Elevated levels of endorphins have been shown to mitigate stress and anxiety, induce relaxation, and regulate circadian rhythms, ultimately enhancing the quality of sleep (Ye et al., 2022). Additionally, Wu’s study suggests that exercising less than three times per week does not significantly improve sleep quality (Wu et al., 2015). Therefore, engaging in physical activity four times per week may yield more favorable results in terms of sleep quality.

This study demonstrates that an exercise duration of ≤30 min is most effective for enhancing sleep quality, aligning with previous research (Wu et al., 2015). Izci-Balserak suggests that, prolonged exercise sessions exceeding 60 min may result in significant muscle fatigue and elevated lactate levels (Izci-Balserak et al., 2022), hastening lymphocyte apoptosis and potentially causing discomfort and lengthening recovery periods, ultimately disrupting nighttime sleep patterns. As exercise duration extends beyond 90 minutes, it may adversely affect psychological and physical health, leading to excessive arousal at night, which can impact sleep quality. (Medic et al., 2017; Chekroud et al., 2018). On the other hand, brief bouts of exercise lasting between 10–30 min have been shown to stimulate the secretion of hormones, including endorphins, which can enhance the quality of sleep by increasing the amount of deep and rapid eye movement sleep (Chan et al., 2019). Other studies have demonstrated that engaging in a 30 min exercise regimen can lead to notable improvements in mental well-being (Tanaka et al., 2002). Maintaining a positive mental state before bedtime has been linked to reduced cognitive arousal prior to sleep onset (Dressle et al., 2023), facilitating quicker and more restful sleep (Lowe et al., 2019; Fank et al., 2022). Moreover, engaging in physical activity for less than 30 min can prevent the prolonged elevation of cortisol levels that may result from extended exercise sessions (Chan et al., 2019). This is significant as heightened cortisol secretion has been linked to disturbances in sleep patterns, including reduced deep sleep stages and disrupted sleep continuity and depth (Athanasiou et al., 2023). Consequently, limiting exercise duration to 30 min or less is shown to be more beneficial in enhancing the quality of sleep.

The study concluded that an exercise intervention lasting 9–10 weeks is most effective in enhancing sleep quality, aligning with previous research findings. A meta-analysis further supports the notion that short-term (<3 months) exercise interventions can notably improve sleep quality (Xie et al., 2021). Hasan’s study suggests that an exercise intervention lasting a minimum of 8 weeks is necessary for significant improvements in sleep quality (Hasan et al., 2022). Moreover, research findings indicate that a 9-week exercise regimen has been linked to notable enhancements in sleep quality, subjective sleep perception, and reduced sleep onset latency. These improvements in subjective sleep quality have been observed to persist for up to 3 months following the conclusion of the exercise intervention (Chan et al., 2014). Additionally, a 10-week exercise program has been demonstrated to effectively decrease sleep fragmentation and elevate melatonin levels, thereby promoting quicker sleep onset (Cai et al., 2014; Bonardi et al., 2016). It is important to note that engaging in exercise for a year may actually lead to difficulties in falling asleep (Tworoger et al., 2003). Thus, a moderate exercise intervention period is more beneficial for enhancing sleep quality.

The results of this study indicate that high-intensity exercise has the best effect on sleep quality. This is inconsistent with existing research results, where meta-analysis results have shown that moderate-intensity exercise interventions have the most significant effect on improving sleep quality (Zhao et al., 2023). This variation may arise from the distinct physiological effects of exercise intensity on the body. Lower exercise intensities have a limited stimulating impact on the sympathetic nervous system (SNS) (Daniela et al., 2022), which is insufficient to induce a significant transition from the SNS to the parasympathetic nervous system (PNS), a shift that is advantageous for enhancing deep sleep and overall sleep quality during the night (Garbarino et al., 2019). Conversely, high-intensity exercise elicits a more pronounced activation of the SNS; following exercise, the heightened SNS activation prompts a robust shift toward PNS activity (Druică et al., 2018), a mechanism that significantly promotes the onset of deep sleep (Trinder, 2012). Additionally, high-intensity exercise has been found to reduce daytime sleepiness (Chekroud et al., 2018), facilitate falling asleep at night (Xie et al., 2022), and decrease the number of awakenings after sleep onset (Larsen et al., 2019), ultimately leading to an overall improvement in sleep quality (Frimpong et al., 2021). Thus, high-intensity exercise is more conducive to the improvement of sleep quality.

At present, systematic comparative studies on the impact of exercise type and dosage combinations on sleep quality effects are still insufficient. In addition, previous studies have often used passive control methods in the setting of control groups, which may to some extent limit the comprehensiveness and accuracy of the results. In light of this, future research should focus on constructing an analytical framework based on different combinations of exercise types and dosages, delving into the specific effects of various types of exercise and their dosage changes on sleep quality, in order to scientifically evaluate the effectiveness of exercise interventions in enhancing sleep quality and to identify the optimal exercise type and dosage combination strategies. At the same time, to strengthen the rigor of research design, it is recommended to use active control methods to effectively reduce the potential impact of the placebo effect, ensuring the reliability and universality of research conclusions. By precisely analyzing the effects of exercise types and dosages on sleep quality, a basis can be provided for developing personalized and precise exercise intervention programs.

This network meta-analysis further supports the efficacy of exercise interventions in enhancing sleep quality. Based on the findings, we recommend the continuation of exercise routines. The research indicates that combined exercises performed four times a week, at a high intensity, over a period of 9–10 weeks, with each session lasting ≤30 min, are more effective in improving sleep quality. We hope that the results of this study will provide evidence for healthcare professionals, educators, and policymakers to make informed decisions. Subsequent research should prioritize the design of exercise types and dosages based on individual attributes, including gender and health status, to enhance the effectiveness of strategies for improving sleep quality.

Several limitations exist for this NMA: ① This study encompassed 58 randomized controlled trials with a total of 5,008 participants; however, the distribution of sample sizes across various types of exercise, exercise periods, durations, intensities, and frequencies were uneven. This disparity somewhat hinders the validity of the statistical analysis. ② The study predominantly evaluated sleep quality using the Pittsburgh Sleep Quality Index, a subjective self-assessment method that may introduce bias into the measurement results. ③ Our research centered on assessing the impact of exercise on sleep quality and did not involve a comparison with alternative intervention measures, such as cognitive behavioral therapy. ④ Furthermore, numerous randomized controlled trials lacked comprehensive descriptions of their randomization procedures, potentially compromising the generalizability and reliability of the study’s conclusions. ⑤ The control groups in the randomized controlled trials selected for this study all employed passive control, which is insufficient to completely rule out the presence of Hawthorne/Placebo effects.

## 5 Conclusion

This study systematically integrates 58 RCTs and analyzes the effectiveness of various exercise programs in improving sleep quality through a network meta-analysis approach. The research focuses on the perspective of exercise type and dosage combinations, comprehensively analyzing these elements of exercise programs to identify the optimal exercise program to improving sleep quality. The results show that a combined exercise program with a frequency of four times per week, a duration of ≤30 min, a period of 9–10 weeks, and a high-intensity level exhibits superior effectiveness in improving sleep quality. This finding transcends the limitations of previous studies that often focused on a single exercise element or short-term exercise effects, and by thoroughly analyzing all five exercise dimensions, it helps to determine a more effective exercise program for improving sleep quality. However, due to the limitations in the number and quality of included literature, as well as potential selection biases, caution should be exercised in interpreting the results of this study. Therefore, future research should further expand the sample size, optimize study designs, and notably in the selection of control groups, active controls should be adopted to effectively control the interference of placebo effects, thereby enhancing the reliability of the research findings.

## Data Availability

The original contributions presented in the study are included in the article/Supplementary material, further inquiries can be directed to the corresponding authors.
