# Supplementary material for: Optimal exercise dose and type for improving sleep quality: a systematic review and network meta-analysis of RCTs
Source: Front Psychol. 2024 Oct 3;15:1466277. doi: 10.3389/fpsyg.2024.1466277 (PMC11484100; doi:10.3389/fpsyg.2024.1466277)
Supplement: Supplementary file 1 [file Table_1.DOCX]

Database search strategy:

Table 1 Research retrieval methods based on Web of Science.

| steps | Search strategies |
| --- | --- |
| #1 | TS=(exercise OR physical activity OR sport OR tai chi OR combined exercise OR aerobic exercise OR walking OR pilates OR yoga OR qi gong OR resistance exercise) |
| #2 | TS=(insomnia OR sleep disorder OR sleep complaint OR sleep disturb OR sleep quality OR sleep problem) |
| #3 | TI=(intervene OR Randomized Controlled Trial OR RCT OR experiment OR trial) |
| #4 | A#1 AND #2 AND #3 |

TS=(exercise OR physical activity OR sport OR tai chi OR combined exercise OR aerobic exercise OR walking OR pilates OR yoga OR qi gong OR resistance exercise) AND

TS=(insomnia OR sleep disorder OR sleep complaint OR sleep disturb OR sleep quality OR sleep problem) AND

TS=(intervene OR Randomized Controlled Trial OR RCT OR experiment OR trial)

Table 2 Search strategies of Pub Med

| steps | Search strategies |
| --- | --- |
| #1 | ("exercise"[Mesh] OR "physical activity"[Mesh] OR "sport"[Mesh] OR "tai chi"[tiab] OR "combined exercise"[tiab] OR "aerobic exercise"[tiab] OR "walking"[Mesh] OR "pilates"[tiab] OR "yoga"[Mesh] OR "qi gong"[tiab] OR "resistance exercise"[Mesh]) |
| #2 | ("insomnia"[Mesh] OR "sleep disorder"[Mesh] OR "sleep complaint"[tiab] OR "sleep disturb"[tiab] OR "sleep quality"[Mesh] OR "sleep problem"[tiab]) |
| #3 | (interven[Title/Abstract]) OR (Randomized Controlled Trial[Title/Abstract]) OR (experiment[Title/Abstract]) OR (trial[Title/Abstract])  ("insomnia"[Mesh] OR "sleep disorder"[Mesh] OR "sleep complaint"[tiab] OR "sleep disturb"[tiab] OR "sleep quality"[Mesh] OR "sleep problem"[tiab]) |
| #4 | #1 AND #2 AND #3 |

("exercise"[Mesh] OR "physical activity"[Mesh] OR "sport"[Mesh] OR "tai chi"[tiab] OR "combined exercise"[tiab] OR "aerobic exercise"[tiab] OR "walking"[Mesh] OR "pilates"[tiab] OR "yoga"[Mesh] OR "qi gong"[tiab] OR "resistance exercise"[Mesh]) AND ("insomnia"[Mesh] OR "sleep disorder"[Mesh] OR "sleep complaint"[tiab] OR "sleep disturb"[tiab] OR "sleep quality"[Mesh] OR "sleep problem"[tiab]) AND ("intervene"[tiab] OR "Randomized Controlled Trial"[Publication Type] OR "RCT"[tiab] OR "experiment"[tiab] OR "trial"[tiab])

Table 3 Search strategies of Embase

| steps | Search strategies |
| --- | --- |
| #1 | ('exercise' OR 'physical activity' OR 'sport' OR 'tai chi' OR 'combined exercise' OR 'aerobic exercise' OR 'walking' OR 'pilates' OR 'yoga' OR 'qi gong' OR 'resistance exercise') |
| #2 | ('insomnia' OR 'sleep disorder' OR 'sleep complaint' OR 'sleep disturb' OR 'sleep quality' OR 'sleep problem') |
| #3 | ('intervene' OR 'Randomized Controlled Trial' OR 'experiment' OR 'trial') |
| #4 | A#1 AND #2 AND #3 |

('exercise' OR 'physical activity' OR 'sport' OR 'tai chi' OR 'combined exercise' OR 'aerobic exercise' OR 'walking' OR 'pilates' OR 'yoga' OR 'qi gong' OR 'resistance exercise') AND ('insomnia' OR 'sleep disorder' OR 'sleep complaint' OR 'sleep disturb' OR 'sleep quality' OR 'sleep problem') AND ('intervene' OR 'Randomized Controlled Trial' OR 'experiment' OR 'trial')

Table 4 Search strategies of Cochrane Library

| steps | Search strategies |
| --- | --- |
| #1 | exercise OR physical activity OR sport OR "tai chi" OR "combined exercise" OR "aerobic exercise" OR walking OR pilates OR yoga OR "qi gong" OR "resistance exercise" |
| #2 | insomnia OR "sleep disorder" OR "sleep complaint" OR "sleep disturb" OR "sleep quality" OR "sleep problem" |

(exercise OR "physical activity" OR sport OR "tai chi" OR "combined exercise" OR "aerobic exercise" OR walking OR pilates OR yoga OR "qi gong" OR "resistance exercise")

AND

(insomnia OR "sleep disorder" OR "sleep complaint" OR "sleep disturb" OR "sleep quality" OR "sleep problem")

After the search, use the built-in filters to limit the results to display only RCTs.

Table 5 Search strategies of Scopus

| steps | Search strategies |
| --- | --- |
| #1 | (TITLE-ABS-KEY(exercise) OR TITLE-ABS-KEY("physical activity") OR TITLE-ABS-KEY(sport) OR TITLE-ABS-KEY("tai chi") OR TITLE-ABS-KEY("combined exercise") OR TITLE-ABS-KEY("aerobic exercise") OR TITLE-ABS-KEY(walking) OR TITLE-ABS-KEY(pilates) OR TITLE-ABS-KEY(yoga) OR TITLE-ABS-KEY("qi gong") OR TITLE-ABS-KEY("resistance exercise")) |
| #2 | (TITLE-ABS-KEY(insomnia) OR TITLE-ABS-KEY("sleep disorder") OR TITLE-ABS-KEY("sleep complaint") OR TITLE-ABS-KEY("sleep disturb") OR TITLE-ABS-KEY("sleep quality") OR TITLE-ABS-KEY("sleep problem")) |
| #3 | (TITLE-ABS-KEY(intervene) OR TITLE-ABS-KEY(RCT) OR TITLE-ABS-KEY(experiment) OR TITLE-ABS-KEY(trial)) |
| #4 | A#1 AND #2 AND #3 |

(TITLE-ABS-KEY(exercise) OR TITLE-ABS-KEY("physical activity") OR TITLE-ABS-KEY(sport) OR TITLE-ABS-KEY("tai chi") OR TITLE-ABS-KEY("combined exercise") OR TITLE-ABS-KEY("aerobic exercise") OR TITLE-ABS-KEY(walking) OR TITLE-ABS-KEY(pilates) OR TITLE-ABS-KEY(yoga) OR TITLE-ABS-KEY("qi gong") OR TITLE-ABS-KEY("resistance exercise"))

AND

(TITLE-ABS-KEY(insomnia) OR TITLE-ABS-KEY("sleep disorder") OR TITLE-ABS-KEY("sleep complaint") OR TITLE-ABS-KEY("sleep disturb") OR TITLE-ABS-KEY("sleep quality") OR TITLE-ABS-KEY("sleep problem"))

AND

(TITLE-ABS-KEY(intervene) OR TITLE-ABS-KEY(RCT) OR TITLE-ABS-KEY(experiment) OR TITLE-ABS-KEY(trial))
